# Back to the Future: Lessons Learned From the 1918 Influenza Pandemic

**DOI:** 10.3389/fcimb.2018.00343

**Published:** 2018-10-08

**Authors:** Kirsty R. Short, Katherine Kedzierska, Carolien E. van de Sandt

**Affiliations:** ^1^School of Chemistry and Molecular Biosciences, The University of Queensland, Brisbane, QLD, Australia; ^2^Australian Infectious Diseases Research Centre, The University of Queensland, Brisbane, QLD, Australia; ^3^Department of Microbiology and Immunology, The Peter Doherty Institute for Infection and Immunity, University of Melbourne, Parkville, VIC, Australia; ^4^Department of Hematopoiesis, Sanquin Research and Landsteiner Laboratory, Amsterdam, Netherlands

**Keywords:** influenza, pandemic, 1918, host factors, viral factors, external factors, societal factors, prevention

## Abstract

2018 marks the 100-year anniversary of the 1918 influenza pandemic, which killed ~50 million people worldwide. The severity of this pandemic resulted from a complex interplay between viral, host, and societal factors. Here, we review the viral, genetic and immune factors that contributed to the severity of the 1918 pandemic and discuss the implications for modern pandemic preparedness. We address unresolved questions of why the 1918 influenza H1N1 virus was more virulent than other influenza pandemics and why some people survived the 1918 pandemic and others succumbed to the infection. While current studies suggest that viral factors such as haemagglutinin and polymerase gene segments most likely contributed to a potent, dysregulated pro-inflammatory cytokine storm in victims of the pandemic, a shift in case-fatality for the 1918 pandemic toward young adults was most likely associated with the host's immune status. Lack of pre-existing virus-specific and/or cross-reactive antibodies and cellular immunity in children and young adults likely contributed to the high attack rate and rapid spread of the 1918 H1N1 virus. In contrast, lower mortality rate in in the older (>30 years) adult population points toward the beneficial effects of pre-existing cross-reactive immunity. In addition to the role of humoral and cellular immunity, there is a growing body of evidence to suggest that individual genetic differences, especially involving single-nucleotide polymorphisms (SNPs), contribute to differences in the severity of influenza virus infections. Co-infections with bacterial pathogens, and possibly measles and malaria, co-morbidities, malnutrition or obesity are also known to affect the severity of influenza disease, and likely influenced 1918 H1N1 disease severity and outcomes. Additionally, we also discuss the new challenges, such as changing population demographics, antibiotic resistance and climate change, which we will face in the context of any future influenza virus pandemic. In the last decade there has been a dramatic increase in the number of severe influenza virus strains entering the human population from animal reservoirs (including highly pathogenic H7N9 and H5N1 viruses). An understanding of past influenza virus pandemics and the lessons that we have learnt from them has therefore never been more pertinent.

## Introduction

In 1918 a mysterious and deadly disease spread around the world in three consecutive waves (spring 1918, autumn 1918, and winter 1918–19). This pandemic infected over one third of the world's population and killed an estimated 50 million people (Johnson and Mueller, 2002; Murray et al., 2006), with unusually severe clinical manifestations in previously healthy young adults (Collins, 1931; Hoffman, 2011). In 1918, the etiological agent that caused this disease was unknown (Hildreth, 1991). However, we now know that these events represented the largest influenza virus pandemic on record: the catastrophic 1918 influenza pandemic. Since 1918, the world has experienced three additional influenza pandemics: the 1957 “Asian” influenza pandemic, the 1968 “Hong Kong” influenza pandemic and the 2009 so-called “swine flu” pandemic. These pandemics, although mild in comparison to that of 1918, highlight the constant threat that influenza virus poses to human health. Given that almost 100 years have passed since 1918, it behooves us to ask: are we truly better prepared for the next influenza virus pandemic or are there still lessons to be learned? This review gives an overview of lessons learned from the 1918 influenza pandemic, highlighting new insights into our understanding of viral pathogenesis and their impact on our preparedness for the next outbreak of influenza.

### The origins 1918 influenza virus

The 1918 influenza pandemic is often colloquially referred to as the “Spanish” influenza pandemic. However, it is unlikely that the 1918 influenza virus originated in Spain. Instead, influenza cases were widely reported in Spain due to the fact that, as a neutral country in World War I, Spain did not practice censorship in the press. In contrast, other countries involved in the war, such as Germany, Britain and France, most likely limited the news of this deadly pandemic, so as not to lower the moral of the troops and raise questions about their military readiness (Johnson, 2006). Today, the general consensus is that the 1918 influenza virus originated in the Midwest of the United States of America (Barry, 2004). Medical records reported the first cases of “influenza of a severe type” around March 1918 in military camps in Kansas (Barry, 2004). From here, the virus is thought to have spread throughout the United Stated and then transported by American troop ships to the battlefields of France, where it gradually spread throughout Europe and the rest of the world (Patterson and Pyle, 1991). The spread of the virus beyond port cities was further facilitated by local transport networks, predominately railways (Patterson and Pyle, 1991; Johnson, 2006). However, it is possible that the predecessor of this killer virus first entered human population prior to 1918 and became more virulent and/or more transmissible over time. Unusual influenza activity was already reported in the United States and several European countries before the first (spring) wave of the 1918 influenza outbreak (Frost, 1919; Johnson, 2006; Hoffman, 2011). Military camps in France already reported influenza infections accompanied with high mortality in the winter of 1916–17 (Hammond et al., 1917), which was followed 2 months later by a similar outbreak near London at Aldershot, one of Britain's biggest military camps (Oxford et al., 1999, 2002, 2005). Interestingly, no records of civilian influenza cases around that time exist, possibly because influenza cases were not recorded at the time or because they got lost with time. Alternatively, it is tempting to speculate that military camps, with their high population density, close proximity to livestock, high mobility, and large number of people with pre-existing lung conditions (due to exposure to toxic gasses in the trenches) served as the perfect breeding ground for the emergence of this catastrophic pandemic (Oxford et al., 2005).

**Figure 1:**
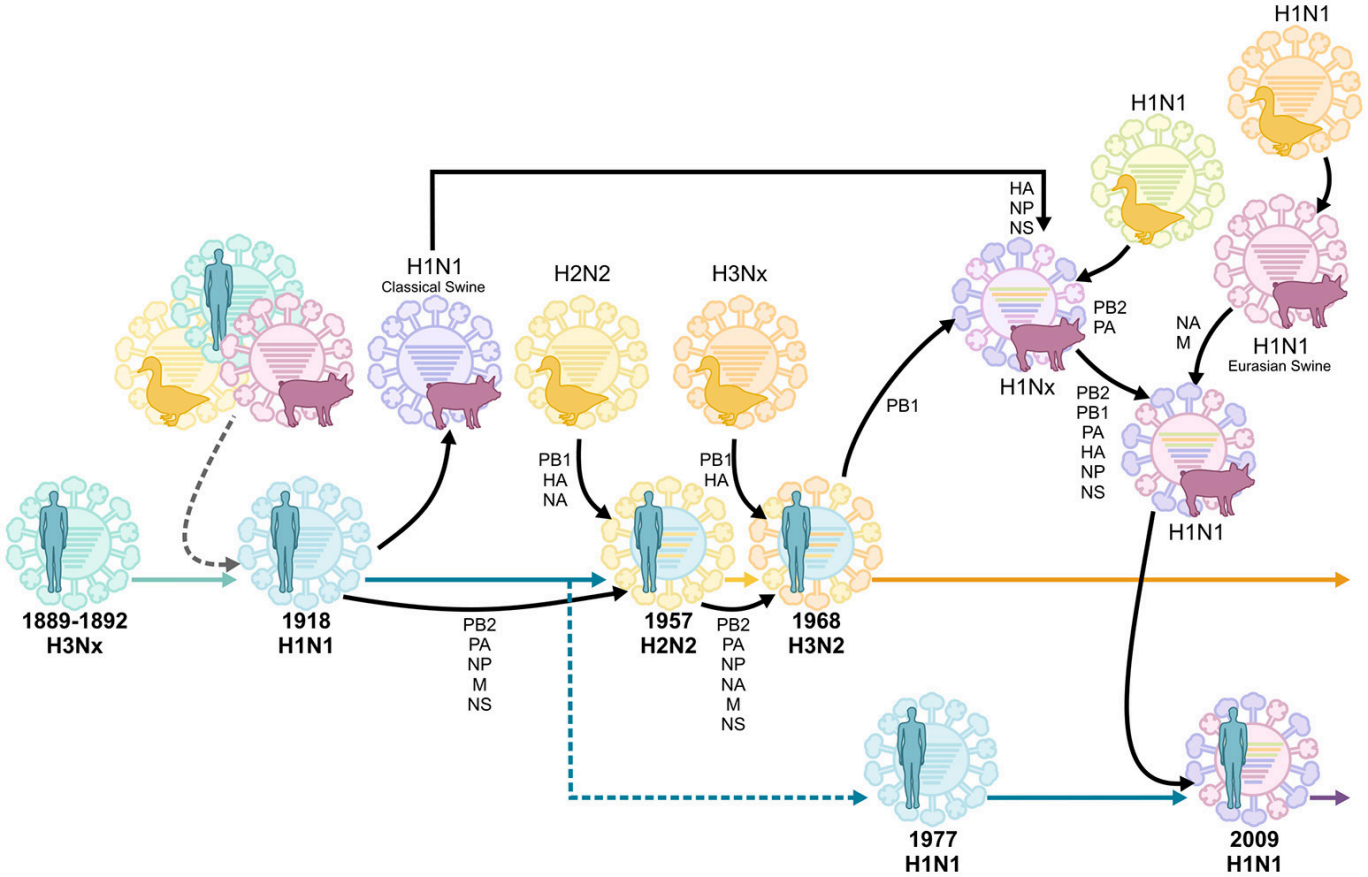
Reassortment events of historic pandemic influenza A viruses, adapted from van de Sandt et al. (2015b). Historic serum analysis suggests that the Russian influenza pandemic of 1889–1892 was of the H3Nx subtype and seasonally circulated up to the 1918 influenza pandemic. It remains undefined whether the 1918 H1N1 pandemic virus originated from multiple reassortment events between avian, swine and human influenza viruses, or if it was introduced by a direct zoonotic transmission event of an avian, swine or other influenza virus. The H1N1 virus continued to circulate, causing seasonal epidemics, until 1957 when it reassorted with an avian H2N2 virus. This virus circulated until 1968, when it reassorted again with the avian H3Nx virus, which has caused seasonal epidemics ever since. In 1977 the H1N1 virus was reintroduced in the human population and co-circulated with H3N2 viruses until the influenza pandemic of 2009 when it was replaced by another H1N1 virus which was the result of multiple reassortment events between avian, swine, and human influenza viruses.

Just as the geographic origins of the 1918 virus remain unclear, the original animal reservoir of the virus also remains controversial. As a segmented virus, influenza virus is capable of undergoing the process of reassortment. Reassortment occurs when two influenza virus strains co-infect the same cell, facilitating the emergence of a new “reassortant” virus which contains a novel constellation of genes. Reassortment between avian and human influenza viruses gave rise to the 1957 and 1968 influenza pandemics (Figure 1; Scholtissek et al., 1978; Kawaoka et al., 1989; Schäfer et al., 1993). In contrast, the 2009 influenza pandemic resulted from a reassortment event between avian, human and swine influenza viruses (Figure 1; Garten et al., 2009; Smith et al., 2009b). Unlike these more recent influenza pandemics, the 1918 virus is thought to have been directly introduced in the human population (i.e., in the absence of reassortment) from a single unidentified host (Taubenberger et al., 2005). This notion is supported by the fact that the 8 individual gene segments of the 1918 virus appear to have co-evolved in the same host. However, the exact identity of this host remains unclear, as the nucleotide sequence of the virus is genetically distinct to all other known avian and mammalian influenza viruses (Reid et al., 2004a,b; Taubenberger et al., 2005). In contrast, others argue that the 1918 influenza virus could have indeed originated from a reassortment event between avian and mammalian, possibly swine and/or human, influenza viruses in the years prior to the 1918 pandemic (Smith et al., 2009a; Worobey et al., 2014). Unfortunately, in the absence of influenza virus sequence data in the years preceding the 1918 pandemic, this question may never be definitively answered.

### A broad spectrum of clinical disease

During the 1918 influenza pandemic, a broad spectrum of clinical illness was observed (Brundage and Shanks, 2008). In the first spring wave of the pandemic, disease was typically mild and mortality rates were not unusually high (Johnson and Mueller, 2002). However, there was a surprisingly large number of young adults who were affected by the outbreak (Ahmed et al., 2007). The second or autumn wave of influenza emerged in late August 1918 and by the end of 1918 almost no country was spared (Patterson and Pyle, 1991; Johnson, 2006). The striking feature of the autumn wave was its unprecedented virulence (Taubenberger et al., 2001). Patients typically suffered from a high fever, cyanosis, and fluid accumulation in the lungs (Johnson, 2006). In ~5% of the fatal cases, death occurred rapidly after the onset of clinical symptoms (i.e., within 3 days), although for the majority of cases the time from clinical symptoms to death was ~7–10 days (Brundage and Shanks, 2008). The third and final wave of influenza emerged at the start of 1919 (Beveridge, 1977). This wave was typically not as virulent as the fall wave and it did not affect every country (Beveridge, 1977; Taubenberger and Morens, 2006). During the course of the pandemic, ~500 million people worldwide were infected, resulting in a case-fatality rate of >2.5% (Johnson and Mueller, 2002; Johnson, 2006; Taubenberger and Morens, 2006). While this fatality rate was certainly higher than other influenza virus pandemics (Taubenberger and Morens, 2006), these data indicate that influenza virus infection was not always fatal and that a large number of people survived the infection. These data raise two intriguing questions: (i) Why was the 1918 influenza pandemic more virulent than other influenza pandemics of the twentieth century; and (ii) Why did some people survive the pandemic and others succumb to the infection?

## Viral factors associated with the severity of the 1918 influenza pandemic

It was not until 1933, more than a decade after the devastating pandemic of 1918–19, when the influenza virus was first isolated and demonstrated to be the causative agent of seasonal influenza virus infections (Smith et al., 1933). However, even then, an in-depth understanding of the viral factors that contributed to the severity of the 1918 pandemic was thwarted by the absence of any available biological material from the virus in question. Finally, in the late 1990s the virus' genetic material was successfully isolated from formalin-fixed, paraffin-embedded lung tissue from 1918 influenza victims and from the lungs of a 1918 influenza victim buried in Alaska's permafrost (Taubenberger et al., 1997; Reid et al., 1999). These efforts unraveled a partial viral sequence from four viruses and the complete genomic sequence of one virus (Reid et al., 1999). The fully reconstructed 1918 influenza virus proved to be highly pathogenic in mice (Tumpey et al., 2005), ferrets (Memoli et al., 2009), and macaques (Kobasa et al., 2007). Interestingly, a recent study in ferrets demonstrated that the 1918 influenza virus could spread to, and induce cytokine responses in tissues outside the respiratory tract, which likely contributed to the severity of the infection (de Wit et al., 2018) and could explain the neurological complications observed during the 1918 influenza pandemic (Alexander, 1919; Ravenholt and Foege, 1982). Various reverse genetics experiments suggest that the high pathogenicity exerted by the 1918 influenza virus was most likely an interplay between different virulence factors, in which proteins encoded by the viral haemagglutinin (HA) and polymerase gene segments played a crucial role (Kobasa et al., 2004; Tumpey et al., 2005; Kash et al., 2006; Conenello et al., 2007; Pappas et al., 2008; Watanabe et al., 2009; Jagger et al., 2010).

One of the best-known virulence determinants of influenza virus is the presence of a multibasic cleavage site in the HA (Horimoto and Kawaoka, 1994; Subbarao et al., 1998). In avian species, influenza viruses without a multibasic cleavage site require the HA to be cleaved by host trypsin-like proteases for infection. Trypsin-like proteases are commonly found in the respiratory tract, thus limiting replication of these viruses to these tissues. However, the presence of the multibasic cleavage site means that these viruses can be cleaved by ubiquitously expressed proteases. The presence of a multibasic cleavage site in modern highly pathogenic avian influenza viruses can be associated with increased virulence in mammalian hosts (Schrauwen et al., 2012; Suguitan et al., 2012). However, none of the available 1918 HA sequences contained a multibasic cleavage site (Kawaoka and Webster, 1988; Taubenberger et al., 1997; Reid et al., 1999). Instead, analysis of the HA sequence of the 1918 viruses revealed that these viruses were adapted to bind to human epithelial cells. The influenza A virus HA protein requires only one amino acid change in order to switch binding to α-2,3-linked sialic acids (typically found on avian cells) to binding to the α-2,6-linked sialic acids (typically found on human epithelial cells in the upper respiratory tract) (Glaser et al., 2005). Compared to victims from the spring wave, an increased incidence of this mutation in the HA was observed in viruses isolated from victims of the more severe autumn wave (Reid et al., 2003; Glaser et al., 2005; Sheng et al., 2011). A second mutation, which strengthens the virus binding to the human receptor, could only be found in some of the 1918 HA sequences (Reid et al., 2003; Sheng et al., 2011). These data suggest that at least two H1N1 influenza viruses circulated in 1918, which differed in their binding affinity for the human receptor. Both viruses displayed a similar cell tropism in the respiratory tract of terminal stage human influenza victims (Sheng et al., 2011). However, this secondary adaptation is essential for effective transmission of the 1918 influenza virus between ferrets (Tumpey et al., 2007). Mutations in other gene segments, including PB2, PA, and PB1-F2 of the 1918 influenza virus can also play a role in host adaptation (Dunham et al., 2009; Jagger et al., 2010; Mehle et al., 2012; Mazel-Sanchez et al., 2018). In the absence of additional influenza virus sequences from 1918, it is hard to establish whether these or other mutations contributed to the dramatic increase in case-fatality seen during the autumn wave of the pandemic (Simonsen et al., 2018).

Gain- and loss-of-function experiments, such as those described above, have provided important insights as to how novel influenza viruses adapt to the human population (Subbarao et al., 1993; Mehle and Doudna, 2009; Herfst et al., 2012; Imai et al., 2012; Belser et al., 2013; Richard et al., 2013; Zhu et al., 2013; Watanabe et al., 2014). Specifically, this information is used to evaluate the pandemic potential of novel influenza viruses, including avian H7N9 and H5N1 viruses, which are frequently crossing the species barrier into the human population. The Global Influenza Surveillance and Response System (GISRS), a surveillance program that monitors which influenza virus strains circulate at a given time (Hay and McCauley, 2018; World Health Organization, 2018a), uses this information for a rapid risk assessment when a potentially pandemic virus is reported to circulate in animals (predominately birds or swine) or has crossed the species barrier into the human population. It is hoped that extensive surveillance activities, in combination with rapid clinical diagnosis, will afford us a “head start” in the case of a future influenza pandemic. However, the success of such surveillance programs is contingent upon their geographical breadth (Krammer et al., 2018). International cooperation and support for influenza surveillance will become even more pertinent in the future as climate changes continues to affect animal reservoirs and avian migration patterns, both of which could lead to the spread of influenza viruses to new locations and across a wider range of avian species (Klaassen et al., 2012; Shaman and Lipsitch, 2013; Audubon, 2018).

## Host factors associated with variations in influenza morbidity and mortality in 1918

The 1918 influenza pandemic is notorious for its high morbidity and mortality rates. However, it is important to recognize that there were substantial variations in mortality, both within and between countries (Mills, 1986; Johnson and Mueller, 2002; Johnson, 2006). General estimations assume an overall death rate of 2.5–5 per 1,000 individuals worldwide. Although this might be an accurate estimate for some countries [e.g., Australia (2.8/1000), Austria (3/1000), Demark (4.1/1000)], it represents an overestimation for some countries [e.g., Argentina (1.2/1000), Uruguay (1.4/1000), American Samoa (0/1000)], and a gross underestimation of others [e.g., Nauru (160/1000), Western Samoa (236/1000), Cameroon (445/1000)] (Johnson and Mueller, 2002; Johnson, 2006). These data indicate that in addition to viral factors, host factors had a major impact on the outcome of infection.

### Age

An individual's age played a major role in determining one's risk of death during the 1918 influenza pandemic. Typically, when the mortality rates of seasonal influenza are graphed against the age of the population, a “U” shaped curve is produced, as the highest mortality occurs in the very young and old (Johnson, 2006). In contrast, pandemic outbreaks (to various degrees) are characterized by a shift in case-fatality toward younger age groups (Simonsen et al., 1998; Olson et al., 2005; Ahmed et al., 2007; Georgantopoulos et al., 2009). This was particularly pronounced during 1918 pandemic when young adults (15–30 years) displayed such usually high mortality rate that a “W” shaped mortality curve was produced (Olson et al., 2005; Ahmed et al., 2007; Shanks and Brundage, 2012). The underlying mechanisms driving this mortality shift are not fully understood but are likely to be associated with the host's immune status.

### Immunopathology

The high mortality rates observed in young adults during the 1918 pandemic has traditionally been attributed to the induction of an aberrant, dysregulated pro-inflammatory response (often referred to as a “cytokine storm”). This hypothesis is based upon experimental studies in various animal models using the reconstructed 1918 influenza virus. These experimental studies showed that the 1918 influenza virus triggered a potent, dysregulated pro-inflammatory response, which likely contributed to the severe lung lesions observed in victims of the 1918 influenza pandemic (Kobasa et al., 2004, 2007; Kash et al., 2006; Memoli et al., 2009; de Wit et al., 2018). Indeed, this dysregulated immune response has also been observed in natural and experimental infections with both the highly pathogenic avian H5N1 virus and the 2009 pandemic influenza virus (de Jong et al., 2006; To et al., 2010). However, it is important to note that all experimental 1918 influenza virus studies to date have been performed in immunologically-naïve animals. This is not necessarily indicative of the human situation in 1918, as influenza viruses caused epidemics and pandemics prior to 1918 (Dowdle, 1999; Johnson, 2006; Morens and Fauci, 2007; Morens et al., 2009; Valleron et al., 2010). It can therefore be assumed that a large proportion of the human population in 1918, with the possible exception of isolated countries/communities, would have encountered at least one previous influenza virus infection, resulting in pre-existing humoral and cellular immunity. It remains unclear whether such pre-existing immunity would cross-react with the 1918 H1N1 virus, and if so, whether it would enhance or dampen any dysregulated pro-inflammatory response in young adults.

### Humoral immune response

In contrast to young adults, older adults (aged 30–60 years) fared significantly better during the 1918 pandemic (Luk et al., 2001). This observation is likely to reflect the beneficial effects of pre-existing humoral immunity. It is theorized that an H1 and/or N1 influenza virus circulated in the human population prior to 1889, when it was replaced by a H3 influenza virus that caused the so-called “Russian” influenza pandemic (1889–1892) (Ahmed et al., 2007). Accordingly, individuals born before 1889 (i.e., those 30 years or older during the 1918 pandemic) would have had cross-protective antibodies, while people born after 1889 would have been immunological naïve to the 1918 H1N1 pandemic virus (Dowdle, 1999; Ahmed et al., 2007). The lack of pre-existing 1918 influenza virus-specific or cross-reactive antibodies in children and young adults likely contributed to the high attack rate and rapid spread of the virus (Ahmed et al., 2007). Only people infected during the first “spring” wave of the pandemic acquired a protective immune response against the second, more virulent, “fall” wave of the 1918–19 pandemic (Gibbon, 1919; Shope, 1958; Palmer and Rice, 1992; Barry et al., 2008; Mathews et al., 2010; Shanks et al., 2010, 2011b; Fraser et al., 2011). Interestingly, unlike the majority of elderly populations worldwide, elderly populations in remote settings, including Indigenous Australians, Alaskan Natives, and Latin Americans, experienced high mortality during the 1918 pandemic. This most likely reflects the fact that these remote populations were not exposed to the previously circulating influenza viruses that conferred cross-protection (Ahmed et al., 2007).

Conclusive evidence that protective influenza virus-specific antibody responses are indeed long-lived came from the 2009 influenza pandemic. Here, elderly people who were exposed to the 1918 influenza virus (or its immediate descendant), 60–90 years prior to the pandemic of 2009, were protected from infection and severe disease, as they maintained the antibody response that cross-reacted with the 2009 pandemic strain (Yu et al., 2008; Hancock et al., 2009; Ikonen et al., 2010; Reed and Katz, 2010).

Interesting, a recent study suggested that individuals medically-treated for influenza-like illness in the years prior to the 1918 pandemic (1916–1918) were actually at an increased risk of having clinically significant respiratory illness during the autumn wave of the 1918 pandemic (Shanks et al., 2016a). Similarly, the presence of cross-reactive but non-neutralizing antibodies, was associated with immune complex deposition and increased disease severity during the 2009 influenza pandemic (Monsalvo et al., 2011). These data suggest that protection against disease is dependent not just upon the presence of pre-existing antibodies, but rather their ability to neutralize the influenza virus strain in question.

### Cellular immune response

The issue of prior influenza virus exposure in the general population prior to 1918 raises the question as to why a pre-existing cellular immune response, in particular cross-reactive CD8^+^ T cells, offered so little protection to young adults during the 1918 influenza pandemic?

A robust CD8^+^ T cell response plays an important role in protection against novel influenza virus strains and subtypes. Unlike antibodies, CD8^+^ T cells can recognize the internal proteins of influenza virus. Since these internal proteins do not undergo rapid antigenic change, CD8^+^ T cells are able to provide cross-protection against a broad range of different influenza virus strains. Accordingly, pre-existing influenza virus specific CD8^+^ T cells provided protection against severe disease during the influenza pandemics of both 1957 and 2009 (Slepushkin, 1959; McMichael et al., 1983; Epstein, 2006; Sridhar et al., 2013; Hayward et al., 2015). In addition, seasonally induced influenza virus-specific CD8^+^ T cells can cross-react with novel potentially pandemic avian influenza viruses (Kreijtz et al., 2008; Lee et al., 2008; van de Sandt et al., 2014) and facilitate more rapid recovery in patients following infection with low pathogenic H7N9 avian influenza virus (Wang et al., 2015). The presence of conserved CD8^+^ T cell peptides in the viral protein sequences of the 1918 influenza virus (Quiñones-Parra et al., 2014) and the ability the of the 2009 H1N1 pandemic influenza virus to recall influenza virus-specific CD8^+^ T cells, cross-reacting with the 1918 H1N1 influenza virus (Gras et al., 2010) suggest that pre-existing CD8^+^ T cells should have been protective against severe infection with the 1918 H1N1 influenza virus, especially in the case of young adults. CD8^+^ T cells may not have been optimal in very young children (age 0–4 years) due lack of exposure to previous influenza viruses (Bodewes et al., 2011a; Sauerbrei et al., 2014). Similarly, immunosenescence may have impaired CD8^+^ T cell function in the elderly (>65 years) (Goronzy and Weyand, 2013). This may partially explain the high mortality observed in the youngest and oldest age groups during seasonal epidemics (U-shaped curve). However, individuals between 15 and 65 years of age, who suffered the greatest burden of disease during the 1918 pandemic, are thought to display the “gold-standard” immune response, with optimal cross-reactive CD8^+^ T cell responses. The absence of protective immunity in this age group is unlikely to be due to the fact that heterosubtypic immunity is short-lived (Mathews et al., 2010), as the longevity of influenza virus-specific CD8^+^ T cells in healthy individuals has recently been demonstrated (van de Sandt et al., 2015a). It is possible that the recall of pre-existing influenza virus-specific CD8^+^ T cell responses was not rapid enough for the extremely virulent 1918 pandemic virus, causing rapid appearance of clinical disease and death within 3 days (Ahmed et al., 2007). Alternatively, pandemic H1N1 influenza viruses (1918 and 2009) may have suppressed immunogenic RIPK3-driven dendritic cell death needed for the induction of an effective CD8^+^ T cell response (Hartmann et al., 2017). In addition, it is likely that ethnically defined genetic variations in HLA molecules influenced cross-reactive CD8^+^ T cell responses in influenza virus infected individuals (Quiñones-Parra et al., 2014). This (combined with other socio-economic factors) would leave some ethnicities, like Alaskan Natives and Indigenous Australians, more susceptible to severe influenza virus infections. Indeed, alarmingly high morbidity and mortality rates were observed amongst these populations during the pandemics of 1918 (Ahmed et al., 2007) and 2009 (La Ruche et al., 2009; Flint et al., 2010). Similarly, it is striking to note that the matrix protein of the 1918 virus already contained extra-epitopic amino acid residues that were associated with evasion from the pre-existing influenza virus CD8^+^ T cells (van de Sandt et al., 2015b), a phenomenon not observed in the comparatively mild 2009 pandemic influenza virus (van de Sandt et al., 2018a,b).

Finally, it is important to note that the highest influenza virus infection rates in 1918 were observed among children of school age (5–15 years). However, this increased infection rate occurred in the absence of high morbidity (Shanks and Brundage, 2012; Mamelund et al., 2016). Thus, school-aged children are thought to be in a “honeymoon period” of superior immunity, whereby they display increased resistance to a variety of different bacterial and viral pathogens (Ahmed et al., 2007). However, the mechanisms underlying such superior immunity are completely unexplored, despite the fact that it holds key information for inducing effective immune responses to influenza viruses.

In this review, we would like to propose an additional hypothesis that might have influenced the effectiveness of the cross-reactive cellular immune response and possibly contributed to the disproportional mortality amongst young adults in 1918; namely immune suppression as a resulting from recent measles infections (Moss et al., 2004; Griffin, 2010; de Vries et al., 2012; Mina et al., 2015). Measles epidemics were frequently reported at the end of the nineteenth and in the early twentieth century (Cliff et al., 1983; Duncan et al., 1997; Shulman et al., 2009; Shanks et al., 2011a, 2014), including a large measles outbreak in the US military camps in the winter of 1917–1918 (Shanks et al., 2014; Morens and Taubenberger, 2015). The elderly population would have experienced measles in their childhood and their immunity would have protected them from contracting measles in the years prior to the 1918 influenza pandemic. However, children and young adults, without prior measles infections, would have been immunologically susceptible to measles in the years preceding the 1918 influenza pandemic. Recent studies have demonstrated that the measles virus infects memory T lymphocytes, resulting in apoptosis and a prolonged state of immune suppression up to 3 years after the initial measles infection (Moss et al., 2004; Griffin, 2010; de Vries et al., 2012; Mina et al., 2015). Influenza virus-specific CTL responses could have been suppressed in young individuals who had to endure a measles infection in the years prior to the 1918 influenza pandemic, which may have increased their susceptibility to a severe influenza infection. The combination of recovering from immunosuppression and an infection with an unexpectedly highly virulent virus might have contributed to severe inflammatory related pathology in a mechanism better known as the immune reconstitution inflammatory syndrome (IRIS) (Hirsch et al., 2004; Morens and Fauci, 2007; Shulman et al., 2009; Barber et al., 2012). Whether recent measles infections indeed led to immunosuppression of the influenza virus-specific T cell responses, resulting in a higher susceptibility for severe influenza virus infections and potential IRIS or alternatively contributed to dampening CD8^+^ T cell immunopathology remains an important area of future research. Fortunately, measles vaccines are now widely available and have greatly reduced the prevalence of measles worldwide (Moss and Griffin, 2012; Perry et al., 2014; Mina et al., 2015). However, the increased number of measles outbreaks in recent years and declining vaccination rates represent a key point of concern for future influenza virus pandemics.

Together, these data demonstrate that an individual's age (and the associated differences in their immune response) played an important role in determining disease outcome in the context of pandemic influenza virus infections. In 2009, age and pre-existing humoral immunity were taken into account when identifying priority individuals for vaccination. In 2009, the elderly population were less susceptible to severe influenza (Dawood et al., 2012) as they were protected through cross-reactive antibodies and CD8^+^ T cells acquired during previous seasonal infections, including the antigenically related A/H1N1 virus that circulated prior to 1957 (Yu et al., 2008; Hancock et al., 2009; Ikonen et al., 2010). Based on these findings, the first limited 2009 influenza vaccine stocks were administered to younger individuals, instead of being misdirected to the traditional high risk group: the elderly (National Center for Respiratory Diseases, CDC, and Centers for Disease and Prevention (CDC), 2009). Improving cross-reactive CD8^+^ T cell responses to influenza vaccinations and natural infections remains a key research priority for the future (Clemens et al., 2018). This includes an understanding of CD8^+^ T cell functionality in ethnically diverse populations and different age groups (Clemens et al., 2018).

### Genetic differences

In addition to the role of humoral and cellular immunity, there is a growing body of evidence to suggest that individual genetic differences contribute to differences in the severity of influenza virus infections. For example, during the 2009 influenza pandemic several single-nucleotide polymorphisms (SNPs) were strongly associated with severe pneumonia. These included SNPs in the genes for interferon response factor 7 (Ciancanelli et al., 2015), Fc fragment of immunoglobulin G, low-affinity IIA, receptor (Zúñiga et al., 2012), RPA interacting protein (Zúñiga et al., 2012), complement component 1q subcomponent binding protein (Zúñiga et al., 2012), CD55 (Zhou et al., 2012), IL-1α (Liu et al., 2013), IL-1β (Liu et al., 2013), surfactant protein B gene (To et al., 2014) and interferon induced transmembrane protein 3 (Everitt et al., 2012; Zhang et al., 2013), and IRF9 (Hernandez et al., 2018). Unfortunately, there is insufficient information available to conclude whether mortality variations in 1918 were influenced by any of the aforementioned SNPs. Defining which SNPs confer increased susceptibility to severe influenza remains an important aspect of influenza pandemic preparedness, as it will help to inform which populations are most at risk of severe disease.

### Malnutrition

Host nutritional status has long been recognized as an important factor in the outcome of a variety of different infectious diseases (Cohen, 2000). In India in 1918, the effects of malnutrition and famine on influenza severity were particularly pronounced. The 1918 influenza pandemic hit India during a widespread drought, which affected the viability of many important food crops (Mills, 1986). Consequently, many Northern-Western, Western and Central Indian provinces experienced a famine during 1918 (Mills, 1986). It was these provinces which also experienced the highest 1918 influenza mortality rates (Mills, 1986). Due to the unusual age distribution of the pandemic, those who succumbed to the disease were typically young adults who formed the majority of the agricultural labor force (Mills, 1986). The resultant labor shortage only served to exacerbate the severity of the influenza pandemic (Mills, 1986). The exact mechanisms by which malnutrition and famine increase the severity of influenza remain to be defined. However, experimental studies suggest that not only does malnutrition suppress the host's immune response to influenza virus, but that it may also facilitate the emergence of novel viral variants, which display increased pathogenicity relative to the original parental strain (Beck et al., 2004).

Undernutrition (often exacerbated by ongoing civil conflicts) remains a problem for influenza pandemics of the twenty-first century and beyond. Indeed, chronic malnutrition was thought to have contributed to the high morbidity and mortality seen in Guatemalan children during the 2009 influenza pandemic (Reyes et al., 2010). Climate change may result in crop failures and exacerbate any food shortages in the future. However, in any future influenza virus pandemic, we will face a “double burden” of malnutrition whereby a proportion of the world's population will experience severe disease because of undernutrition and a proportion of the world's population will experience severe disease because of overnutrition. Specifically, it is now well accepted that obesity increases one's risk of being hospitalized with, and dying from, an influenza virus infection (Morgan et al., 2010; Louie et al., 2011; Van Kerkhove et al., 2011). Perhaps of even greater concern is the fact that obesity inhibits both virus-specific CD8^+^ T cell responses and antibody responses to the seasonal influenza vaccine (Sheridan et al., 2012). The challenge for future influenza pandemics is therefore not only to protect those affected by undernutrition (in particularly in light of the growing problem of climate change), but also the growing number of people living with obesity.

## Underlying infections

### Co-infection with bacterial pathogens

Historical autopsy reports and examination of lung tissue sections from 1918 to 19 influenza case material indicated that for a significant number of patients, the cause of death was not primary viral pneumonia (Brundage and Shanks, 2008; Morens et al., 2008; Chien et al., 2009). Instead, these individuals succumbed to a secondary bacterial infection, most commonly pneumonia caused by bacteria such as *Streptococcus pneumoniae, Haemophilus influenzae, Staphylococcus aureus*, and *Streptococcus pyogenes* (Morens et al., 2008). *H. influenzae* was so frequently observed in influenza patients that it was often cited as the cause of the pandemic (and was thus named accordingly) (Hildreth, 1991). The role of secondary bacterial infections during the 1918 pandemic is consistent with epidemiological observations that while influenza virus attack rates in 1918 were similar among soldiers and civilians, mortality rates were much higher amongst newly arrived soldiers (Shanks et al., 2016b). The unhygienic circumstances in the army camps led to frequent bacterial infections, especially amongst immunologically naïve new army recruits. Thus, following an influenza virus infection, new army recruits were more likely to develop a lethal secondary bacterial pneumonia than civilians or long-serving soldiers (Shanks et al., 2010, 2016b). These observations have been echoed by numerous experimental animal studies, showing that co-infection with influenza virus and bacterial pathogens results in increased disease severity compared to infection with either pathogen alone (Brightman, 1935; Glover, 1941; Francis and de Torregrosa, 1945; Harford et al., 1946; Wilson et al., 1947; Short et al., 2012a, 2013). Different mechanisms have been proposed to explain this viral-bacterial synergism (McCullers, 2006; McAuley et al., 2007; Smith et al., 2013; Hrincius et al., 2015). These include, but are not limited to, reduced mucociliary clearance of inhaled bacteria following influenza virus infection, bacterial adhesion to the basement membrane (Morens et al., 2008; Taubenberger et al., 2012; Chertow and Memoli, 2013) and/or sialic acids exposed by influenza virus (McCullers and Bartmess, 2003; Peltola et al., 2005), viral alterations to the host immune response (Navarini et al., 2006; van der Sluijs et al., 2006; Ballinger and Standiford, 2010; Nakamura et al., 2011; Ellis et al., 2015; Lee et al., 2015) and the bacterial inhibition of epithelial cell repair following initial damage by influenza virus infection (Kash et al., 2011). Importantly, experimental studies suggest that influenza viruses not only increases the severity of secondary bacterial infections, but that it also increases the transmission of *S. pneumoniae* (Diavatopoulos et al., 2010; Short et al., 2012b).

In addition to co-infections with bacterial pathogens such as *S. pneumoniae*, chronic bacterial infections, such as those with *Mycobacterium tuberculosis*, contributed to variations in influenza mortality during the 1918 pandemic. For example, data from a Swiss sanatorium during the 1918 pandemic suggested that the risk of influenza death was higher among tuberculosis (TB) patients than non-TB controls (Oei and Nishiura, 2012). Similarly, individuals with TB were 2.2 times more likely to contract the 1918 influenza virus than non-TB individuals living in the same household (Noymer and Garenne, 2000; Noymer, 2011). A synergistic relationship between *M. tuberculosis* and influenza viruses has also been supported by experimental studies (Redford et al., 2014). The predominance of TB amongst young adults in 1918 may have contributed to the striking “W shaped” mortality curve associated with the 1918 influenza pandemic (Oei and Nishiura, 2012).

Severe complications and morbidity as a result of bacterial co-infections were not unique to the 1918 influenza pandemic. Rather, bacterial co-infections were also observed in the influenza pandemics of 1957, 1968 and 2009, albeit to a lesser extent than in 1918 (Oswald et al., 1958; Robertson et al., 1958; Louria et al., 1959; Oseasohn et al., 1959; Chertow and Memoli, 2013; Joseph et al., 2013). In the 2009 influenza pandemic, TB was also identified as a risk factor for the development of severe disease (Morales et al., 2017). Thankfully, unlike in 1918, the severity of bacterial infections during these more recent influenza pandemics was likely minimized by the use of antibiotics, advanced medical care and the availability of bacterial vaccines [such as the pneumococcal polysaccharide and *H. influenzae* type b (Hib) vaccine] (Oswald et al., 1958; Robertson et al., 1958; Louria et al., 1959; Madhi et al., 2004; Wahl et al., 2018). However, as the rate of antibiotic resistance continues to rise and as pathogens such as methicillin-resistant *S. aureus* (MRSA) (Memoli et al., 2008) and multidrug-resistant *M. tuberculosis* (Zumla et al., 2013; Millard et al., 2015) become more common, we potentially face a future where antibiotics will be ineffectual in the treatment of bacterial infections. This will have direct and severe implications for any future influenza virus pandemic (Memoli et al., 2008). It must be considered an urgent priority to not only minimize antibiotic resistance, but also to invest in the discovery of new antibiotics and alternative treatment options for bacterial infections.

### Malaria

In addition to individuals with bacterial co-infections, mortality during the 1918 influenza pandemic was considerably higher amongst malaria-infected individuals (Langford and Storey, 1992; Afkhami, 2003; Shanks, 2015). Although the underlying mechanism is not fully understood, a malaria-induced procoagulant state could play a role in increasing inflammation and subsequent clinical outcome (Shanks, 2015).

Today, chemopreventive strategies have lowered the disease burden associated with malaria and new eradication strategies are being developed. However, malaria still causes significant worldwide morbidity and mortality, there is ever increasing drug resistance and new malaria vaccines have yet to provide long-lasting benefits at a population level (Ashley et al., 2018). Until effective control measures have been developed and implemented, areas endemic for malaria remain at high risk for increased mortality during the next influenza pandemic.

## Non-pharmaceutical interventions

In 1918, a variety of different approaches were employed to limit the spread of influenza virus and treat infected patients. While many of these methods were of little or no avail, they contain important lessons for influenza pandemic preparedness in the twenty-first century.

### Maritime quarantine

In 1918, when the severity of the second wave of influenza became apparent, many countries imposed strict quarantine measures on all incoming ships to try and prevent the spread of influenza (Johnson, 2006). For the most part, these attempts were unsuccessful (Johnson, 2006). Quarantine measures were either implemented too late and the virus was already present within the country or quarantine was breached by infected individuals who were not yet symptomatic (Crosby, 1976; Tomkins, 1992). Thus, countries such as the U.K. and South Africa dismissed maritime quarantine as impractical and ineffectual (Blakely, 2006; Johnson, 2006). However, Australia imposed the maritime quarantine before any victims of the second wave were reported. All arriving vessels had to be cleared by Commonwealth Quarantine Officials before disembarking. This quarantine protected Australia from the second wave of the pandemic until December 1918 when the quarantine was finally breached. Maritime quarantine thus helped to protect Australia from the worst of the pandemic (Crosby, 2003; Johnson, 2006) and indirectly contributed to protecting certain Pacific Islands that depended on Australian supply ships (Shanks et al., 2018).

The most striking example of this was the mortality difference between American Samoa and Western Samoa. A strict maritime quarantine was imposed in American Samoa by the U.S. Governor in 1918 (Shanks and Brundage, 2013). This quarantine prevented influenza from entering the country, and no deaths from the 1918 influenza were ever recorded in America Samoa (Johnson, 2006; Shanks and Brundage, 2013). This was in sharp contrast to the nearby Western Samoa (located ~100 km away), which did not practice strict maritime quarantine (Tomkins, 1992; Shanks and Brundage, 2013). As a result, Western Samoa was infected by the New Zealand supply ship, the *Talune*, and it is estimated that influenza killed more than a quarter of the population (Tomkins, 1992).

Global transportation has experienced a major transformation in the last century, with ships being replaced by the faster and more widely used air travel. The rise of commercial air travel helps explains the rapid global spread of the more modern influenza pandemics of 1957, 1968, and 2009 in the absence of major military troop movements (Rvachev and Longini, 1985; Hufnagel et al., 2004; Khan et al., 2009; Bajardi et al., 2011; Lemey et al., 2014). Accordingly, maritime quarantine is unlikely to play a role in limiting the spread of any future influenza pandemic. However, in 2009 authorities tried to limit the spread of influenza by using the modern-day equivalent of maritime quarantine: airport arrival screening. Unfortunately, analysis of arrival passengers at Sydney airport in 2009 suggested that airport screening had only a sensitivity of 6.67% for detecting influenza-infected patients, while costing ~$50,000 AUD per case detected (Gunaratnam et al., 2014). This limited efficacy likely reflects the fact that individuals infected with influenza virus can be contagious prior to becoming symptomatic (Hollingsworth et al., 2006). Airport arrival screening is therefore unlikely to control the spread of influenza by international air travel. Rather, advancing modeling (e.g., identifying which travel routes are most vulnerable to disease spread) and a variety of different education campaigns (e.g., raising awareness amongst the general public about the risks of traveling if they have been exposed to an infected individual) are likely to play a more significant role in future pandemic preparedness.

### Mass gatherings

In addition to limiting maritime travel, in 1918 most cities implemented simple non-pharmaceutical interventions to restrict the viral spread. These included imposing restrictions on social gatherings where person-to-person transmission could occur. As a result, schools, theaters, churches, and dance halls were closed, while mass gatherings such as weddings and funerals were banned in order to prevent overcrowding (Frost, 1919; Johnson, 2006; Bootsma and Ferguson, 2007; Hatchett et al., 2007). The peak death rate was lower in cities that rapidly implemented these non-pharmaceutical interventions within a few days after the first local cases were recorded, compared to those which waited a few weeks to respond (Bootsma and Ferguson, 2007; Hatchett et al., 2007). The timing when these interventions were lifted also affected the overall mortality (Bootsma and Ferguson, 2007; Hatchett et al., 2007). Thus, while restrictions on gatherings of people helped reduce influenza virus transmission, as soon as these restrictions were relaxed (typically within 2–8 weeks of their implementation) efficient viral transmission recommenced (Hatchett et al., 2007).

Following the outbreak of the 2009 pandemic influenza virus in Mexico, an 18-day period of mandatory school closure was implemented in the greater Mexico City area (Chowell et al., 2011). This was associated with a 29–37% reduction in influenza transmission (Chowell et al., 2011). Similarly, in Hong Kong there was approximately a 25% reduction influenza virus transmission following secondary schools closures from June 11 to July 10, 2009 (Wu et al., 2010). However, just as in 1918, the duration of these intervention strategies affected their efficacy, and there was a dramatic increase influenza activity in 32 Mexican states in the autumn of 2009, a period which coincided with schools opening for the autumn term (Chowell et al., 2011).

### Facemasks and hygiene

Facemasks were a popular preventative measure employed during the 1918 pandemic. While people were unsure of the etiological agent of the pandemic, the consensus was that it was an airborne disease and wearing a facemask would prevent infections (Crosby, 1976). Accordingly, many cities and regions, including Guatemala City, San Francisco, and certain prefectures of Japan, made wearing a facemask in public places obligatory, and special task forces and education campaigns were established to enforce this regulation (Crosby, 1976; Rice and Palmer, 1993; Rice, 2011). However, in order for a facemask to be at least partially effective against influenza virus it must be (i) worn at all times, (ii) properly made and fitted, and (iii) made of appropriate material. The surgical gauze masks of 1918 often failed to meet these criteria (Crosby, 1976). Thus, the mortality rate of Ontario, Canada (where wearing a mask was voluntary) was not significantly different from Alberta, Canada, (where mask wearing was enforced by law) (MacDougall, 2007). In fact, influenza deaths in Alberta continued to rise even after mask wearing was sanctioned by law, suggesting that in 1918 wearing a facemask was not sufficient to prevent deaths from influenza (World Health Organization Writing Group et al., 2006).

Proper hygiene (e.g., frequent hand washing) would also have helped limiting the spread of the influenza virus during the 1918 pandemic, as influenza viruses are transmissible via hand to face contact (World Health Organization Writing Group et al., 2006; Thomas et al., 2014). Thus, the Japanese traditional attitude to disease and illness might have contributed to a lower national pandemic mortality in 1918–19, as Japanese children are taught to remove their shoes and wash their hands upon re-entering the home (Rice and Palmer, 1993).

In the context of modern influenza pandemics, facemasks and handwashing/hand sanitizers have been used as preventative, non-pharmaceutical interventions. However, during the 2009 influenza pandemic for the most part, the use of facemasks was not obligatory (CDC, 2009). Rather, the CDC only recommended facemasks for individuals at increased risk of severe illness from influenza and/or individuals who were the direct careers of persons infected with the pandemic virus (CDC, 2009). Moreover, the effectiveness of facemasks in preventing the transmission of influenza virus remains unclear (Cowling et al., 2010) and just as was observed in 1918, low public compliance significantly limits the utility of facemasks in a modern pandemic setting (Cowling et al., 2010). Perhaps such interventions will be of greatest relevance to medical personal, who serve in the front line of a pandemic and are at high risk for infection. In contrast, handwashing and the use of hand sanitizers (whether or not in combination with wearing a facemask) had a clear protective effect during the influenza pandemic of 2009 (Larson et al., 2012; Suess et al., 2012; Wong et al., 2014).

These data suggest that non-pharmaceutical interventions such as social distancing, handwashing/hand sanitizers, and facemasks in any future influenza pandemic may buy valuable time before vaccines become widely available. However, the success of these interventions will depend upon their early and continuous implementation and also people's willingness to comply. The 1918 pandemic has shown that measures are most effective when they are voluntary, as people have low tolerance for mandatory health measures (Spinney, 2017). Indeed, a behavioral study showed that individuals were more likely to wear a facemask when they received autonomy-supportive advice as compared to controlled instructions (Chan et al., 2015).

## Medical interventions, therapies and vaccines: then and now

The 1918 influenza pandemic occurred during a period in history when controlling infectious diseases had become a realistic goal of the medical profession (Tomkins, 1992; Johnson, 2006). Public health initiatives had already proven successful in limiting the spread of diseases such as cholera and TB (Hildreth, 1991; Tomkins, 1992; Tognotti, 2003). Thus, there was initially little to suggest that an influenza outbreak could not be effectively controlled (Hildreth, 1991; Tomkins, 1992; Tognotti, 2003). However, despite the dramatic advances in microbiology in the previous decades, the etiological agent of the 1918 influenza pandemic remained a mystery. In the absence of clear information about the causative agent of the pandemic, a range of different therapeutic and preventative treatments were attempted. People experimented with medications (including Asprin) and homemade remedies such as mustard poultice, quinine, tobacco, beef tea, the inhalation of zinc sulfate, opium, salt water, and alcohol (Rice and Palmer, 1993; Johnson, 2006; Starko, 2009; Keeling, 2010). Traditional eastern medicine, like the Japanese *Kanpo* medicine (consisting of herbal remedies accompanied by green tea) may have had some beneficial effect by stimulating perspiration (helping to reduce fever), improving vitamin C levels and replacing lost fluids (Palmer and Rice, 1992; Rice and Palmer, 1993). Similarly, the use of Traditional Chinese Medicine may have reduced the severity of influenza infections in at least some individuals (Kobayashi et al., 1999; Cheng and Leung, 2007; Chen et al., 2011). However, for the most part little was available in terms of effective therapeutic and/or prophylactic treatments. Nursing care actually proved to have contributed to the recovery of patients, especially those suffering from secondary bacterial pneumonia (Robinson, 1990; Rice and Palmer, 1993). In addition, mortality rates were significantly higher in places deprived from nursing care, e.g., mining compounds (Phimister, 1973; Rice and Palmer, 1993). Unfortunately, during the 1918–19 pandemic many of those who typically performed these duties were either serving overseas or were sick themselves (Crosby, 2003; Keeling, 2010; Shanks et al., 2011b).

Today, the identification of the etiological agent of influenza has dramatically improved diagnostic speed and accuracy. Rapid and highly accurate molecular diagnostic techniques have largely replaced the labor intensive and time consuming “gold standard” cell culture method for diagnosing influenza virus infections (Ellis and Zambon, 2002), which allows for rapid isolation of infected individuals. Furthermore, risk assessment of potentially pandemic viruses has greatly improved by screening the viral genome of human and animal virus isolates for the presence of mutations that increase human adaptation and/or virulence.

In addition, we are able to deploy both anti-viral drugs and vaccines in the case of an influenza virus pandemic. Antivirals (such as the neuraminidase inhibitors oseltamivir and zanamivir) can be used as a therapeutic in severely ill patients, while also being employed prophylactically in outbreak situations (Cooper et al., 2003; Hayden et al., 2004; De Clercq, 2006; Zambon, 2014; Krammer et al., 2018). At present, potentially pandemic influenza viruses (such as avian H7N9 and H5N1 viruses) are sensitive to both oseltamivir and zanamivir (Herfst et al., 2012). However, acquired resistance to oseltamivir has been observed in several H5N1 isolates (De Clercq, 2006). Similarly, oseltamivir resistance is known to emerge in H7N9 viruses within just 2 days from the start of treatment (Hay and Hayden, 2013). These data suggest that in the case of any future influenza virus pandemic, antivirals should be used judiciously, and the emergence of drug-resistant viral variants closely monitored.

Influenza virus vaccines have also played a major role in reducing the morbidity and mortality associated with seasonal influenza. Unfortunately, antibodies elicited by seasonal influenza vaccines do not provide protection in the case of an antigenically distinct influenza virus of a novel subtype, such as A/H5N1 or A/H7N9 (De Jong et al., 2000). Furthermore, current inactivated seasonal influenza vaccines may even prevent the induction of cross-reactive CD8^+^ T cell responses, which are our primary protection in case of a pandemic outbreak and may therefore prove to be a double-edged sword (Bodewes et al., 2009a,b, 2011b,c). Rapid vaccine production also remains a challenge for future influenza virus pandemics (World Health Organization, 2005; Rockman and Brown, 2010; Pada and Tambyah, 2011). This was particularly apparent during the 2009 pandemic when sufficient amounts of the vaccine against the pandemic virus were only available in October 2009, well and truly after the pandemic had spread globally (Butler, 2010). Vaccine production in a pandemic scenario may be further complicated by the fact that some avian influenza viruses can kill the embryonated chicken eggs needed for vaccine production (Tumpey et al., 2005). Novel vaccines strategies, in combination with alternative vaccine production platforms are needed to accelerate vaccine production and circumvent such problems (Schotsaert and García-Sastre, 2014). However, an influenza vaccine that offers long-lasting, broad-spectrum immunity remains the gold standard for pandemic preparedness. How basic fundamental humoral and cellular biology and human clinical data can be considered for the implementation of a universal influenza vaccine has recently been reviewed (Clemens et al., 2018).

## Concluding remarks

**Figure 2:**
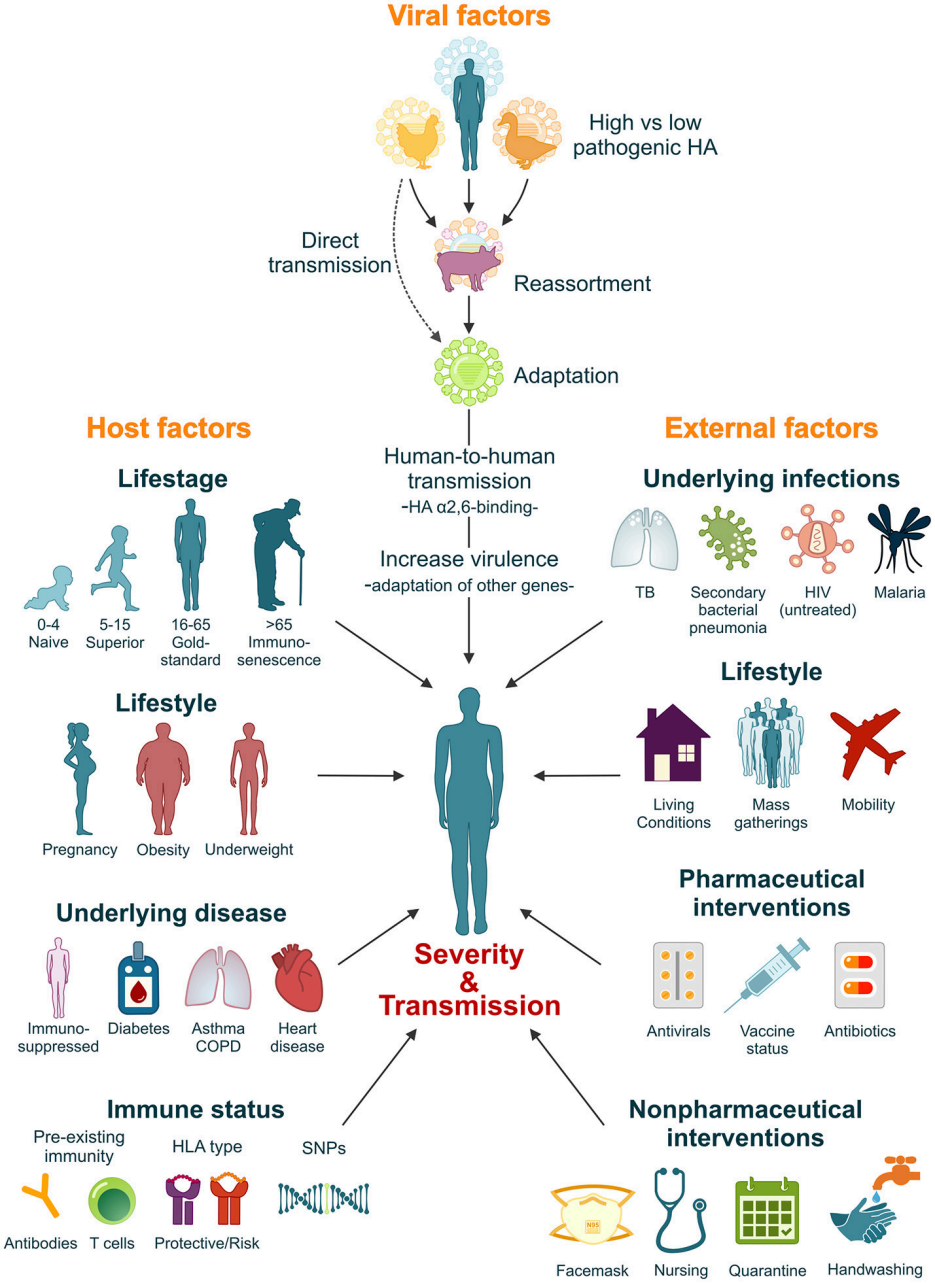
Factors that influence the severity and transmissibility of a pandemic influenza virus. The severity and transmissibility of pandemic influenza viruses are the result of a complex interplay of viral, host, and external factors. We have come a long way since 1918 and pandemic preparedness programs have learned from the 1918 and later pandemic outbreaks. Although unlikely, we cannot exclude the possibility that an influenza pandemic with similar severity will repeat itself in the future. However, lessons learned from the 1918 influenza pandemic will ensure that we are better prepared.

It is estimated that if a pandemic influenza virus were to re-appear today, with a similar virulence and attack-rate as the 1918 influenza virus, mortality could rise to 21–147 million (Murray et al., 2006; Madhav, 2013). However, the high morbidity and mortality rates associated with the 1918 influenza pandemic resulted from a complex interplay between factors intrinsic to the 1918 virus itself, the host's immune response and the social context in which the pandemic struck. It is thus unlikely that this exact combination of factors would repeat itself in the future. Nevertheless, a comprehensive understanding of the factors that contributed to the severity of the 1918 pandemic plays an important role in our preparedness for the next influenza pandemic (Figure 2).

Today, we are better prepared for the next influenza virus pandemic than we were 100 years ago. Global influenza surveillance programs have been established to constantly monitor whether influenza viruses cross the species barrier into the human population (Hay and McCauley, 2018; World Health Organization, 2018a; Ziegler et al., 2018). This has already resulted in improved management strategies (Krammer et al., 2018) and the preventive slaughter of vast numbers of poultry that were infected with potentially pandemic viruses, such as H5N1 and H7N9 viruses (Oi, 2018). In addition, an improved understanding of the host-adaptation of influenza viruses and the existence of pre-existing immunity are likely to contribute to a more accurate predication of viral severity even before the influenza virus in question becomes established as a pandemic (Kreijtz et al., 2008; Lee et al., 2008; Herfst et al., 2012; Imai et al., 2012; Richard et al., 2013; Quiñones-Parra et al., 2014; van de Sandt et al., 2014; Wang et al., 2015). A better understanding of the human immune response against (pandemic) influenza viruses will eventually aid the development of broad-protective influenza vaccines (Clemens et al., 2018). However, in the interim, the majority of countries have established a pandemic preparedness program, which defines the precautionary measurements needed to be taken in case of an emerging viral pandemic (van Genugten et al., 2001; RIVM, 2018; World Health Organization, 2018b). These programs include surveillance, diagnostics, screening of passengers traveling from a potential outbreak region, quarantine procedures, stockpiling antibiotics, antivirals, bacterial and viral vaccines and the distribution of medical supplies (Brundage, 2006; Memoli et al., 2008; Mossad, 2009; World Health Organization, 2018b). We have also learnt from the 2009 H1N1 pandemic that it is important to have a somewhat flexible approach to pandemic preparedness, which allows countries to develop and implement their own risk assessments based on the global assessments provided by the WHO (Rudenko et al., 2015). However, good communication between countries and the WHO remains essential. In addition, it will be important for governments and those in authority to gain public trust before the next major pandemic outbreak. This will ensure that the public knows what to expect, how to act and is likely to improve compliance with preventative measures in a pandemic scenario. The internet represents a powerful and effective tool to disperse such information (Little et al., 2015).

Despite the advances that we have made in pandemic preparedness over the last 100 years, there are also several new challenges that we face in the context of twenty-first century (and later) influenza pandemics. Today's population demographic is dramatically different to that of 1918. Today, a large percentage of the world's population is either elderly (Morens et al., 2008; Mossad, 2009; Murray and Chotirmall, 2015) and/or living with one or more chronic medical conditions [such as heart disease, obesity, asthma, chronic obstructive pulmonary disease (COPD), and/or diabetes mellitus] (Morens et al., 2008; Jain et al., 2009; La Ruche et al., 2009; Flint et al., 2010; Hulme et al., 2017). The number of immunosuppressed individuals (due to untreated HIV infection, transplantation or/and chemotherapy) is also increasing (Jain et al., 2009; Kunisaki and Janoff, 2009; Sheth et al., 2011). This changing population demographic is of significance as each one of these host factors is known to increase the severity of even mild influenza virus infections. Mitigating the severity of future influenza pandemics will be further complicated by the prevalence of antibacterial resistance (Memoli et al., 2008), an increasing negative attitudes toward vaccination for other infectious diseases (such as measles and the pneumococcal polysaccharide and Hib vaccine) (Moss and Griffin, 2012; Perry et al., 2014) and an increase in seasonal influenza vaccinations of healthy individuals affecting the cross-reactive immune response otherwise induced by natural influenza virus infections (Bodewes et al., 2009b, 2011b). The high prevalence of underlying infections in less economically developed countries (such as TB and HIV), coupled with an underprepared health care system, places less economically developed countries at particularly high risk of severe morbidity during future influenza virus pandemics (Murray et al., 2006). These effects may even be compounded by the impacts of climate change which will lead to food shortages, famine and migration of climate refugees (van Schaik and Bakker, 2017).

At present, it is impossible to predict which influenza virus strain will cause the next pandemic. However, the growing number of human infections with the avian H7N9 virus represents a point of concern (especially in light of the approximate 40% mortality rate of this virus in humans). Like previous influenza pandemic viruses, human H7N9 virus infections have thus far displayed multiple waves of infections and shown signs of adaptation to human hosts (Zhu et al., 2017). Although this virus has yet to display efficient human-to-human transmission (Chen et al., 2013), it serves as a timely reminder that even though it has been 100 years since the 1918 pandemic, influenza pandemic preparedness remains of a paramount importance.

## Author contributions

CvdS planned the review. KS, KK, and CvdS wrote the manuscript.

### Conflict of interest statement

The authors declare that the research was conducted in the absence of any commercial or financial relationships that could be construed as a potential conflict of interest.
